# Particulate Matter Elevates Ocular Inflammation and Endoplasmic Reticulum Stress in Human Retinal Pigmented Epithelium Cells

**DOI:** 10.3390/ijerph20064766

**Published:** 2023-03-08

**Authors:** Sunyoung Jeong, Eui-Cheol Shin, Jong-Hwa Lee, Jung-Heun Ha

**Affiliations:** 1 Bioanalytical and Pharmacokinetic Research Group, Korea Institute of Toxicology, Daejeon 34114, Republic of Korea; 2 Department of Human and Environmental Toxicology, University of Science and Technology, Daejeon 34113, Republic of Korea; 3 Department of GreenBio Science/Food Science and Technology, Gyeongsang National University, Jinju 52725, Republic of Korea; 4 Department of Food Science and Nutrition, Dankook University, Cheonan 31116, Republic of Korea; 5 Research Center for Industrialization of Natural Neutralization, Dankook University, Yongin 16890, Republic of Korea

**Keywords:** particulate matter, ocular exposure, inflammation, endoplasmic reticulum stress, unfolded protein response

## Abstract

Because of their exposure to air, eyes can come into contact with air pollutants such as particulate matter (PM), which may cause severe ocular pathologies. Prolonged ocular PM exposure may increase inflammation and endoplasmic reticulum stress in the retina. Herein, we investigated whether PM exposure induces ocular inflammation and endoplasmic reticulum (ER) stress-related cellular responses in human retinal epithelium-19 (ARPE-19) cells. To understand how PM promotes ocular inflammation, we monitored the activation of the mitogen-activated protein kinase (MAPK)/nuclear factor kappa beta (NFκB) axis and the expression of key inflammatory mRNAs. We also measured the upregulation of signature components for the ER-related unfolded protein response (UPR) pathways, as well as intracellular calcium ([Ca^2+^]_i_) levels, as readouts for ER stress induction following PM exposure. Ocular PM exposure significantly elevated the expression of multiple cytokine mRNAs and increased phosphorylation levels of NFκB-MAPK axis in a PM dose-dependent manner. Moreover, incubation with PM significantly increased [Ca^2+^]_i_ levels and the expression of UPR-related proteins, which indicated ER stress resulting from cell hypoxia, and upregulation of hypoxic adaptation mechanisms such as the ER-associated UPR pathways. Our study demonstrated that ocular PM exposure increased inflammation in ARPE-19 cells, by activating the MAPK/NFκB axis and cytokine mRNA expression, while also inducing ER stress and stress adaptation responses. These findings may provide helpful insight into clinical and non-clinical research examining the role of PM exposure in ocular pathophysiology and delineating its underlying molecular mechanisms.

## 1. Introduction

Prolonged exposure to air pollutants poses a threat to human health and the quality of life [1,2]. Numerous epidemiological studies have demonstrated that exposure to airborne particulate matter (PM) is associated with a significant increase in hospitalization and mortality rates [3,4]. In 2013, the International Agency for Research on Cancer classified PM as a Group 1 carcinogen to humans [5]. Moreover, approximately 99% of the global population is exposed to PM concentrations above the safety limit standards for air quality recommended by the World Health Organization. The PM is a mixture of solid and liquid particles suspended in air and consists of various components, such as elemental carbon (soot), organic carbon (including polycyclic aromatic hydrocarbons [PAH], nitro-PAHs, and endotoxins), sulfate, nitrate, and minerals [6]. There are three common categorization groups for PM depending on the aerodynamic diameter of its particles: (1) coarse (PM10; ≤10 μm), (2) fine (PM2.5; ≤2.5 μm), and (3) ultrafine (PM0.1; ≤0.1 μm) particles [7]. Inhaled PM is primarily deposited in the upper respiratory tract, whereas fine particles are delivered to the lower respiratory tract [8,9]. Moreover, ultrafine PM can be delivered into the bloodstream [8]. Therefore, the distribution and toxicity of PM mainly depend on particle size [8]. Exposure to PM has been reported to adversely affect the respiratory [9], cardiovascular [10], renal [11,12], hepatic [13], dermal [14], and nervous systems [15].

PM can also come into contact and affect the visual system, either via the bloodstream, which is initiated by pulmonary inhalation [16] or through the ocular surface [17,18]. Ocular PM exposure causes various types of discomfort to the eye, such as redness, itching, foreign substances, and dryness [19,20]. The cornea and conjunctiva are directly exposed to external toxicants since they are the outermost protective layers of the eye, and are therefore more vulnerable to suspended air particles, compared to posterior regions, such as the retina. Because of these characteristics, dry eye syndrome (DES) and allergic conjunctivitis closely correlate with ocular PM exposure [21,22]. Recent epidemiological studies have supported that PM exposure closely correlates with an increase in retinal, as well as ocular surface diseases. Higher PM exposure has been associated with an increased prevalence of age-related macular degeneration (AMD) in South Korea [23], the UK [24], Canada [25], and Taiwan [26]. Moreover, patients with diabetes who are also chronically exposed to PM, present with a significantly increased risk of diabetic retinopathy (DR) in Taiwan [27] and China [28]. In a cohort study, participants exposed to higher PM and NO_2_ concentrations showed adverse retinal structure features in the UK [29]. Short-term (24 h exposure to PM significantly narrowed retinal vessels, as well as both arterioles and venules in healthy adults [30]. Toxicological studies suggest that acute respiratory PM exposure causes retinal edema, which may be mediated by hypoxic responses [12]. In another rodent-based study, PM exposure in the eye triggered retinal vascular permeability, with retinal edema and inflammation [31]. Long-term PM exposure in the rat eye markedly reduced the thickness of the total retinal layer, including an observed decrease in the thickness of the nerve fiber layer/ganglion cell layer in the retina [32]. At a molecular level, PM treatment increased c-Jun N-terminal kinase (JNK) phosphorylation and led to increased secretion of interleukin-6 (IL-6) and tumor necrosis factor alpha (TNFα) in human retinal pigment epithelium (RPE) cells [33]. Furthermore, ocular PM exposure may interrupt intact ocular function by inducing intracellular production of reactive oxygen species, mitochondrial dysfunction, and enhancing epithelial-mesenchymal transition, in spontaneously arising RPE 19 (ARPE-19) cells [34]. However, a more robust toxicological mechanism is required to understand how ocular PM exposure disrupts ocular homeostasis.

Inflammation is considered one of the main effects of PM exposure in various organs including the eye [15,35,36,37]. In rodent studies, several lines of evidence have supported that topical PM administration on the eye induces symptoms similar to clinical DES [17,21,37,38] or conjunctivitis [18,37]. Currently, only limited publications demonstrate that PM exposure triggers retinal inflammation [31,33]. Topical PM administration (10 μg, four times daily, for two days) reportedly increased inflammatory mRNA expression of genes such as *intercellular adhesion molecule 1* (*Icam1*), *lymphocyte common antigen* (*CD45*), and the *nucleotide-binding oligomerization domain-like receptor family pyrin domain containing 3* (*Nlrp3*), in the rat retina [31]. In addition, PM exposure has also been linked to increased inflammatory protein expression and leukocyte infiltration into retinal vessels [31]. In another study, a localized PM eye drop (2 μg, twice daily, for 21 days) caused a significant degree of myopia, with ocular inflammation in Syrian hamsters, and drastically elevated inflammatory TNFα and IL-6 protein expression in the retina, cornea, and sclera [39]. Similarly to topical PM exposure, whole-body exposure (long-term; six months) can also reportedly induce retinal inflammatory cytokines release in mice, including TNFα and cleaved IL-1β, while concomitantly causing retinal thinning and apoptosis, and ultimately lead to poor response to light stimuli [40].

There is a growing literature that suggests ocular inflammation and endoplasmic reticulum (ER) stress are closely intertwined and are strong pathological triggers for DR [41,42,43,44], AMD [42,43,44,45], and retinitis pigmentosa [42,43,44]. To date, there is no direct association between PM exposure and ocular ER stress. However, PM exposure has been shown to increase unfolded protein responses (UPRs) in other organs. Prolonged PM inhalation induces ER stress with UPR activation in lung and liver tissues of murine models [46], and chronic PM inhalation (10 months) also triggers macrophage infiltration in mouse white adipose tissue in vivo [47]. In addition, whole-body exposure to PM for 3–6 months deteriorates renal function, with autophagy induction, UPRs, and apoptosis in Sprague Dawley rats [48].

Since ocular PM exposure triggers inflammation, we postulated whether it may also increase ocular ER stress and the associated adaptive UPRs. Here, we investigated whether PM exposure induced ocular inflammation and ER stress-related cellular responses in ARPE-19 cells. To examine how PM promotes ocular inflammation, we monitored the mitogen-activated protein kinase (MAPK)/nuclear factor kappa beta (NFκB) axis activation, as well as the expression of inflammatory cytokine mRNAs. Moreover, ocular UPR pathways induction and intracellular calcium ([Ca^2+^]_i_) levels were also monitored, to delineate the mechanism behind PM-dependent retinal ER stress.

## 2. Materials and Methods

### 2.1. PM Preparation

PM (PM_10_-like; European reference material ERM-CZ120) was purchased from Sigma-Aldrich (St. Louis, MO, USA), and resuspended in normal phosphate buffer saline (PBS; 100 mg/mL or diluted as needed), aliquoted, and stored at −20 °C until future use. Suspensions were sonicated (Powersonic 420; Hwasin Technology Co., Seoul, Republic of Korea) for 20 min and vortexed for 1 min before each experiment to minimize aggregation.

### 2.2. Cell Culture

The ARPE-19 cell line (CRL-2302) was purchased from the American Type Culture Collection (Manassas, VA, USA) and cultured in Dulbecco’s modified eagle medium (DMEM/F12; Gibco, Carlsbad, CA, USA) supplemented with 10% fetal bovine serum (FBS; Biowest, Nuaillé, France), and 1% gentamicin (Gibco), at a 5% CO_2_, 37 °C incubator (Vision Scientific Co., Ltd., Daejeon, Republic of Korea).

### 2.3. PM Treatment

The ARPE-19 cells were plated in 6-well plates (SPL Life Sciences Co., Ltd., Pocheon, Republic of Korea) at a density of 5.0 × 10^5^ cells/well and cultured for 48 h. Our experiments were conducted using the following research designs:

- For inflammatory response, cells were exposed to varying doses of PM (0, 50, 100, 250, or 500 μg/mL) for 30 min (for western blot analysis) or 2 h (for qRT-PCR analysis). To understand whether mitogen-activated protein kinase kinase (MEK) is involved in PM-induced ocular inflammation, cells were pretreated for 30 min with a mixture of 20 μM U0126; a selective inhibitor of MEK1/2, and 500 μg/mL PM.
- For ER stress-inducing conditions, cells were treated with 2 mg/mL of PM for 0, 2, 4, 6, and 8 h, while 0.01% Tween-80 was used as vehicle treatment.

### 2.4. Cell Viability Assay

Cell viability was examined by the 3-[4,5-dimethylthiazol-2-yl]-2,5 diphenyl tetrazolium bromide (MTT) assay. The ARPE-19 cells were seeded onto the bottom of a 96-well plate (SPL Life Sciences Co., Ltd.) at a concentration of 2.0 × 10^4^ cells per well. The MTT assay was performed under two different conditions: (1) to determine the cytotoxicity of PM, cells were treated with various doses of PM (0, 25, 50,100, 250, 500, 1000, and 2000 μg/mL, respectively), or PBS as a control, for 2 h, and (2) to examine ER stress-related responses, 0.01% Tween-80 and 2000 μg/mL of PM were co-treated for 4 or 8 h. The MTT solution (1 mg/mL) was then added to each well, followed by incubation at 37 °C for 3 h. The medium was removed carefully and formazan crystals were dissolved in 100 μL dimethyl sulfoxide (DMSO; Daejung Chemicals & Metals Co., Ltd., Siheung, Republic of Korea). Absorbance was measured at 540 nm using a microplate reader. Each condition was replicated across five wells on each plate.

### 2.5. Western Blot Analysis

To extract the total protein, ARPE-19 cells were washed thrice with ice-cold PBS and lysed in radioimmunoprecipitation assay (RIPA) buffer (ATTO, Tokyo, Japan) supplemented with protease and phosphatase inhibitors (Thermo Fisher Scientific, Waltham, MA, USA). The protein concentration was determined using the bicinchoninic acid (BCA) protein assay (Thermo Fisher Scientific, Waltham, MA, USA). Thirty micrograms of protein were loaded and separated on 10% sodium dodecyl sulfate (SDS) polyacrylamide gels, and subsequently transferred onto polyvinylidene difluoride (PVDF) membranes (Bio-Rad Laboratories, Hercules, CA, USA) using a wet-transfer method. Next, blots were blocked with 5% skim milk (BD Difco, Franklin Lakes, NJ, USA) for 1 h at room temperature, and were then incubated with primary antibodies overnight at 4 °C. After washing, the membranes were incubated with secondary horseradish peroxidase (HRP)-conjugated antibodies at room temperature for 1 h. Finally, the blots were developed using a chemiluminescent reagent (Thermo Fisher Scientific, Waltham, MA, USA). The specific bands were visualized on a Davinch Western™ Imaging system (Davinch-K, Seoul, Republic of Korea) and then quantified using ImageJ software version 1.53k (National Institutes of Health, Bethesda, MD, USA). Each band was normalized to the relevant total protein amount of the glyceraldehyde 3-phosphate dehydrogenase (GAPDH), which served as an internal loading control. The primary and secondary antibodies used for western blotting are summarized in Table 1.

### 2.6. Quantitative RT-PCR Analysis

Total RNA was extracted using a NucleoSpin RNA Plus kit (Macherey-Nagel, Düren, Germany) according to the manufacturer’s instructions. Then, 1 μg of total RNA was reverse-transcribed to cDNA using ReverTra Ace™ qPCR RT Master Mix (Toyobo, Osaka, Japan). Real-time PCR was performed using a RealMOD™ Green W^2^ 2x qPCR mix (Intron Biotechnology, Sungnam, Republic of Korea) according to the manufacturer’s protocol. The relative gene expression was normalized to that of *36B4* [49], which did not vary significantly with treatment. The PCR primers used are listed in Table 2. The *ANKRD37* primers were purchased from Bio-Rad Laboratories (unique assay ID: qHsaCID0010971; Hercules, CA, USA).

### 2.7. Intracellular Calcium Release

Cytosolic [Ca^2+^]_i_ was measured using a Fluo-4 NW Calcium Assay kit (F36206; Thermo Fisher Scientific, Waltham, MA, USA) according to the manufacturer’s protocol. The ARPE-19 cells were plated at a density of 2.0 × 10^4^ cells/well in a dark-walled 96-well plate (Corning, Corning, NY, USA) and cultured overnight. The next day, cells were gently washed with HBSS, loaded with 100 μL of Fluo-4 NW dye and incubated at 37 °C for 30 min in the dark. Following the addition of PM, fluorescence was monitored using a microplate reader (Molecular Devices, San Jose, CA, USA).

### 2.8. Statistical Analysis

All data are expressed as means ± standard deviations (SDs). Significant differences were statistically analyzed using one-way analysis of variance (ANOVA), followed by Tukey’s post hoc analysis. Differences were considered statistically significant at *p* < 0.05 and are further indicated by a filled asterisk above each bar. To test the efficacy of U0126, the 500 and 500+U0126 groups were compared by Student’s *t*-test. Statistically significant differences were defined as *p* < 0.05 and are indicated by a filled pound sign above the bar for the 500+U0126 group. All statistical analyses were performed using the GraphPad Prism 5 software (GraphPad Software, San Diego, CA, USA).

## 3. Results

### 3.1. PM Exposure Triggers Ocular Inflammatory Responses in ARPE-19 Cells

**Figure 1:**
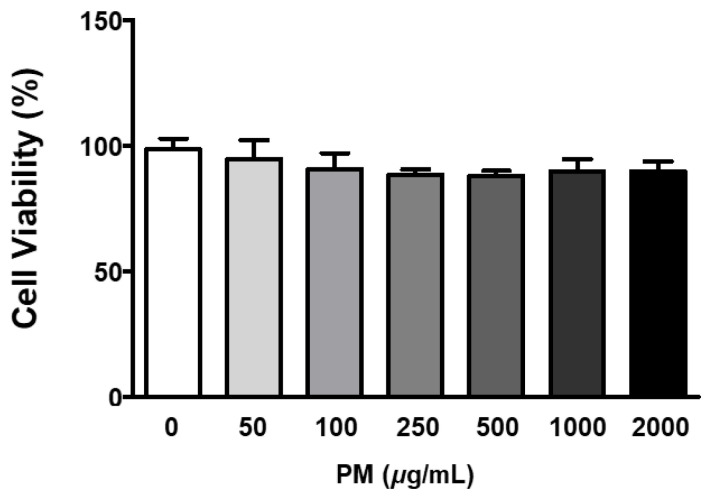
Effect of PM on ARPE-19 cell viability. Cells were treated with 0–2000 μg/mL of PM for 2 h and viability was measured by the MTT assay. The relative ratios to vehicle control are presented as percentages. Data are portrayed as mean ± SD (*n* = 5). Data were analyzed using one-way ANOVA followed by Tukey’s multiple comparison. No significant differences were detected. PM; particulate matter.

**Figure 2:**
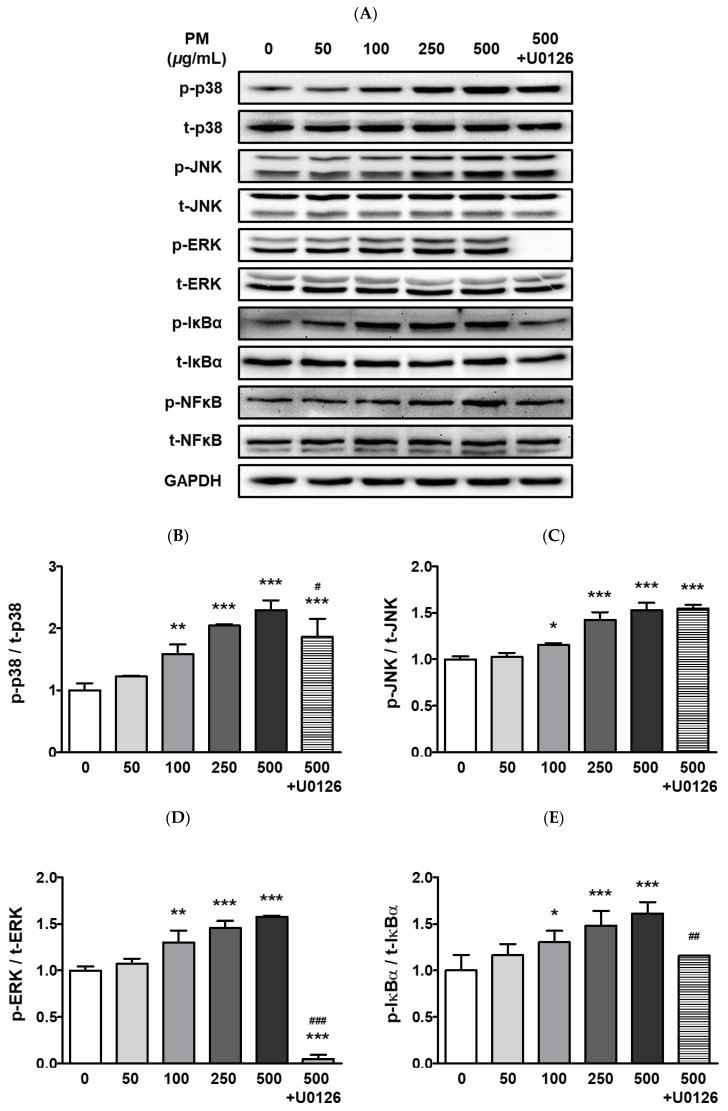

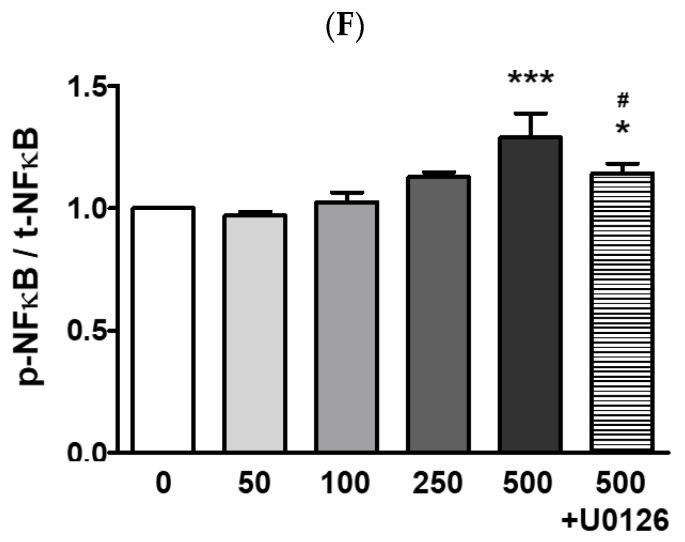
Effect of PM on MAPK and NFκB proteins in ARPE-19 cells. Cells were treated with 0–500 μg/mL of PM for 30 min. U0126 (20 μM) was pretreated for 30 min before PM (500 μg/mL) exposure. (**A**) Phosphorylation levels of p38, JNK, ERK, IκBα, and NFκB were analyzed by western blot. GAPDH was used as the loading control and for normalization. The protein expression ratios of (**B**) p-p38/t-p38, (**C**) p-JNK/t-JNK, (**D**) p-ERK/t-ERK, (**E**) p-IκBα/t-IκBα, and (**F**) p-NFκB/t-NFκB are shown. Data are portrayed as mean ± SD (*n* = 3). Data were analyzed using one-way ANOVA followed by Tukey’s multiple comparison (* *p* < 0.05, ** *p* < 0.01, *** *p* < 0.001). Asterisk signs were used to indicate comparisons against the vehicle control group. Also, data were analyzed by student’s *t*-test and pound signs were used for comparisons between the 500 and 500+U0126 groups (# *p* < 0.05, ## *p* < 0.01, ### *p* < 0.001). PM; particulate matter, MAPKs; mitogen-activated protein kinases; p38; p38 MAPK, JNK; c-Jun N-terminal kinase, ERK; extracellular signal-regulated kinases, NFκB; nuclear factor kappa B p65, IκBα; NFκB inhibitor alpha, GAPDH; glyceraldehyde-3-phosphate dehydrogenase; p; phosphorylated, t; total.

The ARPE-19 cells were treated with 0–2000 μg/mL of PM for two hours, and cellular viability was measured by the MTT assay. Our results indicated that PM did not significantly affect cell viability at any of the tested concentrations (Figure 1). To determine whether PM exposure induced MAPK/NFκB axis-dependent ocular inflammatory responses in ARPE-19 cells, we examined the phosphorylation status of p38 MAPK (p38), JNK, and extracellular signal-regulated kinase (ERK), as well as NFκB inhibitor alpha (IκBα) and NFκB, by western blot analysis. Exposure to PM significantly increased the phosphorylated (p) portion of all MAPKs tested, in a dose-dependent manner (Figure 2). The amount of p-p38 protein significantly increased compared to total (t) p38, after exposure at PM concentrations ≥100 μg/mL, in direct correlation with the PM amount present (Figure 2B). Similarly, phosphorylation of JNK and ERK was also notably enhanced by ocular PM exposure at concentrations ≥100 μg/mL (Figure 2C,D). Posttranslational phosphorylation of MAPKs is closely associated with IκBα degradation and the NFκB’s translocation to the nucleus. As expected, phosphorylation of IκBα and NFκB in human retinal epithelial cells was increased following exposure to PM at doses ≥100 μg/mL and 500 μg/mL, respectively (Figure 2E,F).

**Figure 3:**
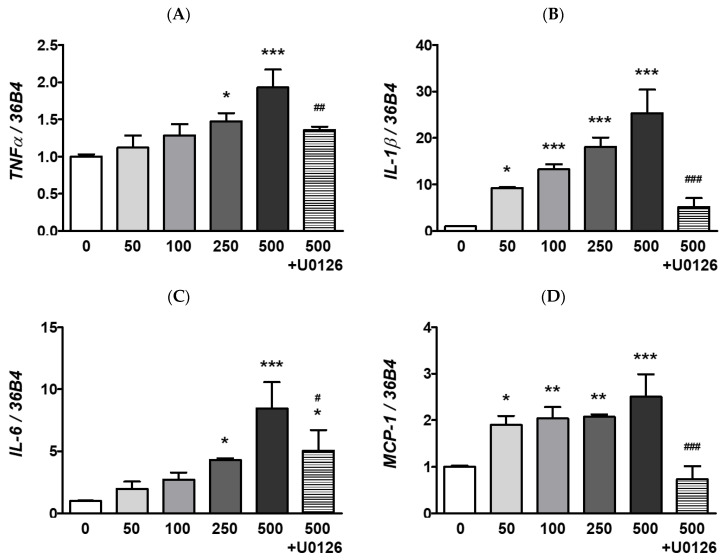
Effect of PM on inflammatory mRNAs expression in ARPE-19 cells. Cells were treated with 0–500 μg/mL of PM for 2 h. The U0126 (20 μM) was applied for 30 min before PM (500 μg/mL) exposure. The mRNA levels of (**A**) *TNFα*, (**B**) *IL-1β*, (**C**) *IL-6*, (**D**) *MCP-1* were measured by qRT-PCR, using *36B4* as the reference gene for normalization. Data are depicted as mean ± SD (*n* = 3). Data were analyzed using one-way ANOVA followed by Tukey’s multiple comparison (* *p* < 0.05, ** *p* < 0.01, *** *p* < 0.001). Asterisk signs were used to indicate comparisons against the vehicle control group. Also, data were analyzed by student’s *t*-test and pound signs were used for comparisons between the 500 and 500+U0126 groups (# *p* < 0.05, ## *p* < 0.01, ### *p* < 0.001). PM; particulate matter, *TNFα; tumor necrosis factor alpha, IL-1β; interleukin-1 beta, IL-6; interleukin-6, MCP-1; monocyte chemoattractant protein-1*.

To identify the potential mechanism of the MAPKs/NFκB axis activation by ocular PM exposure, an ERK inhibitor, U0126 was used prior to PM incubation. The U0126 pretreatment with PM significantly prevented the phosphorylation of p38, ERK, IκBα, and NFκB (Figure 2). This result indicates that activation of the MAPK/NFκB axis by PM exposure likely depends on the phospho-activation of ERK. Moreover, ocular inflammatory responses are regulated not only by posttranslational modifications but also by transcriptional mechanisms. Therefore, we analyzed the mRNA expression of ocular cytokines following PM exposure. As shown in Figure 3, PM treatment significantly elevated expression of the ocular cytokine mRNAs of *TNFα* ([PM] ≥ 250 μg/mL), *IL-1β* ([PM] ≥ 50 μg/mL), *IL-6* ([PM] ≥ 250 μg/mL), and *monocyte chemoattractant protein-1* (*MCP-1*; [PM] ≥ 50 μg/mL) (Figure 3). Similarly to our described results for MAPK/NFκB, ERK inhibitor treatment also significantly attenuated the PM-induced ocular cytokine mRNA expressions (Figure 3). These results indicate that PM promotes ocular inflammation via the activation of NFκB-related inflammatory pathways and upregulation of inflammatory cytokines.

### 3.2. PM Exposure Induces Ocular ER Stress in ARPE-19 Cells

**Figure 4:**
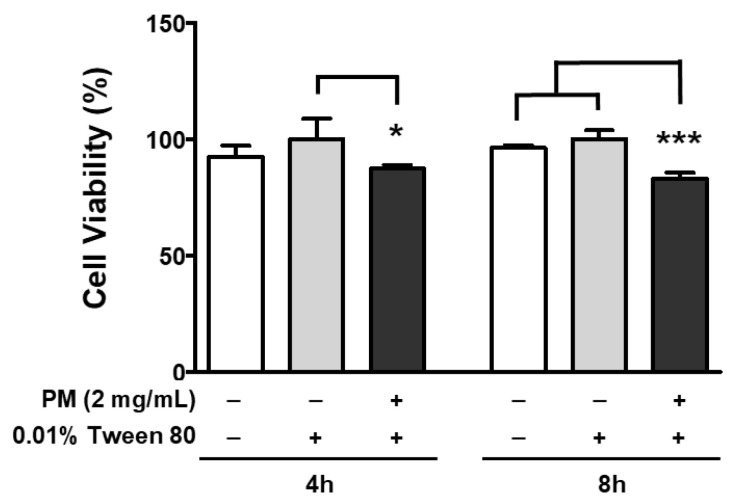
Effect of PM and 0.01% Tween-80 on ARPE-19 cell viability. Cells were treated with 2 mg/mL of PM for 4 or 8 h and viability was measured by the MTT assay. A concentration of 0.01% Tween-80 was used as a vehicle control. The relative ratios to vehicle control are presented as percentages. Data are portrayed as mean ± SD (*n* = 5). Data were analyzed using one-way ANOVA followed by Tukey’s multiple comparison (* *p* < 0.05, *** *p* < 0.001). PM; particulate matter, MTT; 3-(4,5-dimethylthiazol-2-yl)-2,5-diphenyltetrazolium bromide.

To increase the delivery efficacy of PM within cells, the PM was prepared with 0.01% Tween-80 to prevent internal aggregation, and also increase the cellular absorption rate by maintaining a smaller size. Park et al. have reported that PM size is smaller when Tween-80 is used as a vehicle, compared to PM alone [50]. Therefore, Tween-80 as a vehicle control could serve as an efficient means of exacerbating cellular toxicity, by facilitating PM delivery. In our experimental setting, treatment with 0.01% Tween-80 alone did not affect the viability of ARPE-19 cells at any of the time points monitored (4 and 8 h), as this was similar to the untreated control (Figure 4). However, when 2 mg/mL of PM was delivered together with 0.01% Tween-80, retinal cell viability was significantly decreased to 87% and 83% after 4 and 8 h, respectively (Figure 4).

**Figure 5:**
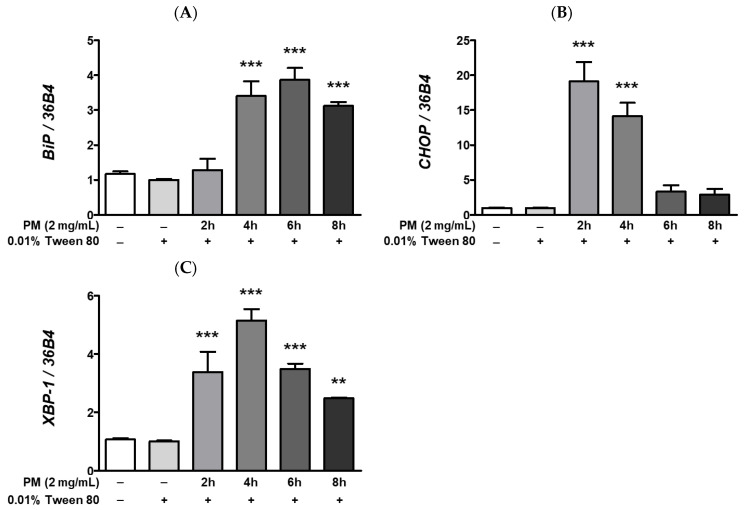
Effect of PM on UPR-related mRNAs expression in ARPE-19 cells. Cells were treated with 2 mg/mL of PM for 0–8 h. An amount of 0.01% Tween-80 was used as a vehicle control. The mRNA levels of (**A**) *BiP*, (**B**) *CHOP*, and (**C**) *XBP-1* were measured by qRT-PCR, using 36B4 as the reference gene for normalization. Data are shown as mean ± SD (*n* = 3). Data were analyzed using one-way ANOVA followed by Tukey’s multiple comparison (** *p* < 0.01, *** *p* < 0.001). All comparisons were made to the vehicle control group. Abbreviations used in Figure: PM; particulate matter, *BiP; binding of immunoglobulin protein, CHOP; CCAAT/enhancer-binding protein homologous protein, XBP-1; X-box binding protein 1*.

Endoplasmic reticulum stress is a strong cellular pathogenic trigger of various metabolic complications. Neurodegenerative disorders such as Alzheimer’s or Parkinson’s disease, diabetes, atherosclerosis, liver disease, cancer [51], and infectious diseases caused by bacteria or viruses [52] have been associated with prolonged ER stress. Exacerbation of ER stress is also a key pathological signature of diverse ocular diseases such as DR [53,54], glaucoma [53], and AMD [53,55]. Therefore, modulation of ocular ER stress is crucial for mitigating or preventing ocular diseases. To investigate whether PM causes ocular ER stress in ARPE-19 cells, we examined the expression levels of the ER stress-related mRNAs; *binding of immunoglobulin protein* (*BiP*), *CCAAT/enhancer-binding protein homologous protein* (*CHOP*), and *X-box binding protein 1* (*XBP-1*), by qRT-PCR. As seen in Figure 5, in line with our hypothesis, PM treatment significantly increased expression of ER stress-related mRNAs compared to our vehicle control (0.01% Tween-80). Expression of *BiP* significantly increased after 4 h of PM treatment, while *CHOP* mRNA expression peaked earlier, at 2 h following ocular PM exposure, in a similar manner to *XBP-1*, whose expression was also markedly elevated at ≥2 h post-PM treatment (Figure 5).

**Figure 6:**
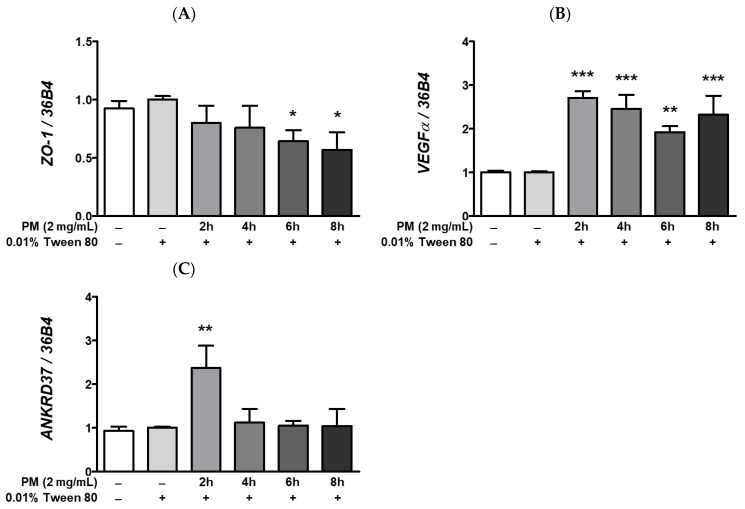
Effect of PM on hypoxia response-related mRNAs expression in ARPE-19 cells. Cells were treated with 2 mg/mL of PM for 0–8 h and 0.01% Tween-80 was used as a vehicle control. The mRNA levels of (**A**) *ZO-1*, (**B**) *VEGFα*, and (**C**) *ANKRD37* were measured by qRT-PCR, using *36B4* as the reference gene for normalization. Data are depicted as mean ± SD (*n* = 3). Data were analyzed using one-way ANOVA followed by Tukey’s multiple comparison (* *p* < 0.05, ** *p* < 0.01, *** *p* < 0.001). All comparisons were made against the vehicle control group. Abbreviations used in Figure: PM; particulate matter, *ZO-1*; *zonula occludens-1*, *VEGFα*; *vascular endothelial growth factor alpha*, *ANKRD37*; *ankyrin repeat domain 37*.

**Figure 7:**
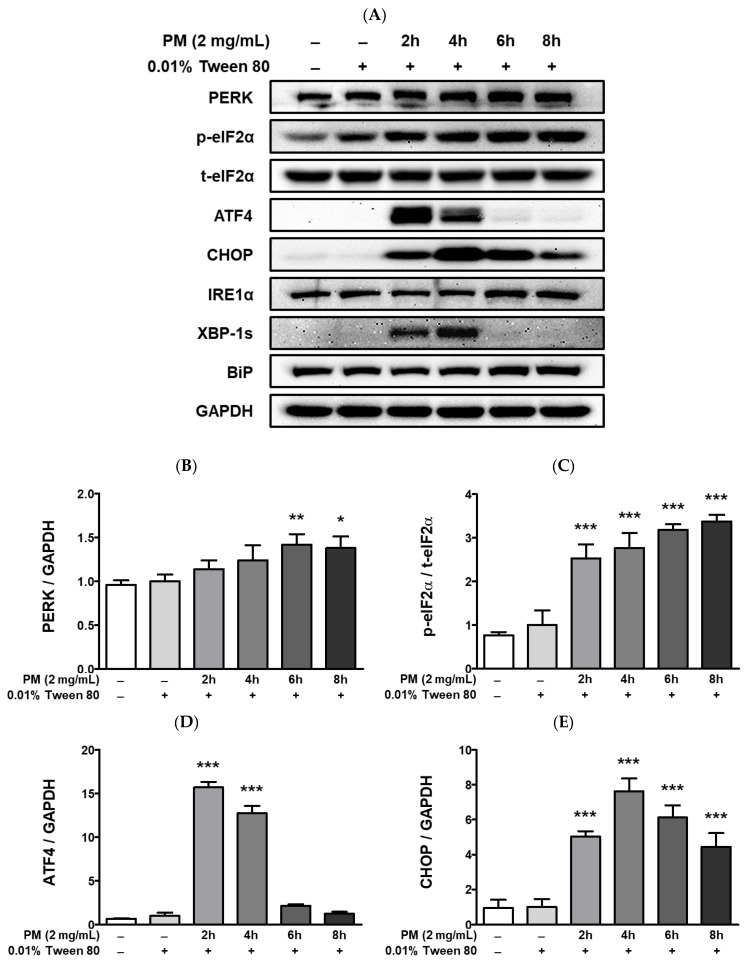

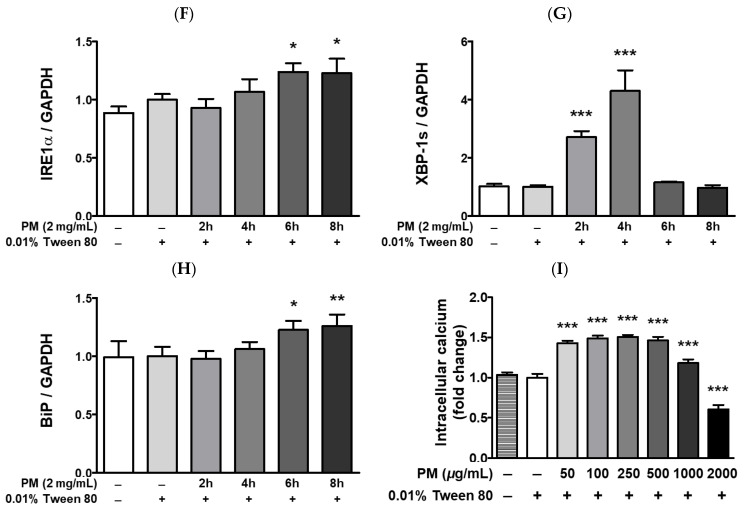
UPR-related protein expression and intracellular calcium levels [Ca^2+^]_i_ in PM-treated ARPE-19 cells. Cells were treated with 2 mg/mL of PM for 0–8 h and (**A**) UPR-related protein expression levels were assessed by western blot, using 0.01% Tween-80 as a vehicle control and GAPDH as the assay loading control and for normalization. The protein expressions of (**B**) PERK, (**C**) p-eIF2α/t-eIF2α, (**D**) ATF, (**E**) CHOP, (**F**) IRE1α, (**G**) XBP-1s, and (**H**) BiP are shown. (**I**) Cytosolic [Ca^2+^]_i_ level was measured by fluorescence. Data are portrayed as mean ± SD (*n* = 3). For calcium measurement; *n* = 5. Data were analyzed using one-way ANOVA followed by Tukey’s multiple comparison (* *p* < 0.05, ** *p* < 0.01, *** *p* < 0.001). All comparisons were made against the vehicle control group. Abbreviations used in Figure: PM; particulate matter, PERK; protein kinase R-like endoplasmic reticulum kinase, eIF2α; eukaryotic translation initiation factor 2 alpha, ATF4; activating transcription factor 4, CHOP; CCAAT/enhancer-binding protein homologous protein, IRE1α; inositol-requiring enzyme 1 alpha, XBP-1s; spliced X-box binding protein 1, BiP; binding of immunoglobulin protein, GAPDH; glyceraldehyde-3-phosphate dehydrogenase, p; phosphorylated, t; total.

Calcium is essential in maintaining the integrity of protein folding and posttranslational modification mechanisms [56], and the ER is a major site of intracellular calcium storage. Moreover, prolonged ocular ER stress is closely correlated with increased [Ca^2+^]_i_ levels and the subsequent induction of hypoxic adaptation responses, including the hypoxia-related UPR. An imbalance in [Ca^2+^]_i_ levels between cytosol and the ER in the eye may induce significant ocular ER stress. Pharmacological induction of ER stress by treatment with the non-competitive sarco/endoplasmic reticulum inhibitor of the Ca²⁺ ATPase, thapsigargin, has been shown to increase [Ca^2+^]_i_ levels and expression of hypoxia-associated mRNAs such as *vascular endothelial growth factor* (*VEGF*), in ARPE-19 cells [49]. Since we observed that PM exposure remarkably elevates expression of ER stress-related transcripts (Figure 5), we posited whether PM may also promote hypoxia and activate hypoxic adaptation responses in retinal cells. In our experimental setting, ocular PM exposure decreased the *zonula occludens-1* (*ZO-1*) mRNA expression in a time-dependent manner (Figure 6A). This observed reduction in *ZO-1* mRNA levels may be related to retinal inflammation (Figure 2 and Figure 3), hypoxia (Figure 6B,C), and UPRs (Figure 5 and Figure 7) following PM exposure. As per our expectations, expression of hypoxia-related mRNAs, *VEGFα* and *ankyrin repeat domain 37* (*ANKRD37*) was remarkably elevated following ocular PM exposure (Figure 6B,C). Both *VEGFα* and *ANKRD37* are the direct target genes of the hypoxia-inducible factor 1 (HIF-1), therefore, their upregulation following PM exposure indicates that PM likely promotes hypoxic responses in retinal cells. Furthermore, induction of the ocular hypoxic response may also be closely related to the disruption of epithelial tight junction integrity [57]. Ocular epithelial tight junctions have been shown to be affected by various stress conditions, including increased inflammation [58], hypoxia [57], and unfolded protein responses [59].

PM treatment elevated cytosolic [Ca^2+^]_i_ levels and lead to the upregulation of hypoxia-related UPRs (Figure 7). Exposure to PM leads to a significant increase in the protein levels of protein kinase R-like endoplasmic reticulum kinase (PERK), eukaryotic translation initiation factor 2 alpha (eIF2a), activating transcription factor 4 (ATF4), and CHOP, as well as of inositol-requiring enzyme 1 alpha (IRE1α), XBP-1, and the ER chaperone BiP (Figure 7A–H). Compared to the vehicle control, [Ca^2+^]_i_ levels in ARPE-19 cells were remarkably elevated after exposure to PM doses between 50–1000 μg/mL (Figure 7I). Interestingly, [Ca^2+^]_i_ levels closely mirrored the gradual increase in PM concentration in a dose-dependent manner up to 250 μg/mL [PM], but exhibited a rapid decrease at PM doses ≥500 μg/mL (Figure 7I). This trend in the fluctuation of cytosolic [Ca^2+^]_i_ may be related to retinal apoptosis, however, further mechanistic studies are required.

## 4. Discussion

The pathological progression of retinal diseases is closely intertwined with significant risk factors such as inflammation and ER stress, which may ultimately threaten vision. Increased ocular inflammation and ER stress have been associated with DR [41,54], and AMD [45]. Progression of ocular diseases cannot be easily identified or predicted in patients, thereby prevention through avoidance of pathological toxicants is highly recommended. Owing to industrial development, carbon-containing fuels (i.e., fossil fuels) are widely used, which emit significant amounts of by-products after combustion, including PM. According to current literature, PM can be a potent inducer of inflammatory [15,35,36] and ER stress responses [46,47,48] in tissues. In this study, we investigated whether ocular PM exposure induces inflammatory responses and ER stress in retinal cells. Our findings demonstrated that treatment with PM induced ocular inflammatory responses in ARPE-19 cells by activation of the MAPK/NFκB axis, with a concomitant upregulation in the expression of inflammatory mRNAs. In addition, ocular PM exposure increased [Ca^2+^]_i_ levels and promoted hypoxia-related UPRs.

The retina plays an important role in vision because it converts incoming light into electrical signals. We employed the ARPE-19 cell line for our experiments since RPE cells have a primary immune defense role by locating the outermost layer of the retina and forming a blood-retinal barrier, thereby contributing to ocular integrity through the maintenance of tight junctions [60]. The RPE also possesses a phagocytic function that eliminates the photoreceptor outer segments, rendering the light-sensing region sensitive to light exposure [60]. Furthermore, the RPE also transports numerous nutrients and eliminates metabolic by-products in the eye [61]. Therefore, abnormalities in RPE cells may cause retinal dysfunction and ultimately lead to the development of vision-related disorders [62].

The TNFα, IL-1β, and IL-6 cytokines are expressed as part of the initial response to inflammation [63]. Moreover, MCP-1 is also a chemokine involved in the inflammatory cascade that regulates the recruitment of monocytes/macrophages to local sites of inflammation [64]. In the present study, ocular PM exposure led to a significant increase in inflammatory responses in ARPE-19 cells, as it induced expression of the cytokine mRNAs *TNFα*, *IL-1β*, *IL-6*, and *MCP-1* (Figure 3). The activation of NFκB-signaling promotes pro-inflammatory cytokine and chemokine production [65,66]. Along with NFκB, MAPK-signaling is mainly activated by cellular stress as well as inflammatory stimuli; therefore, the MAPK/NFκB axis plays an integral role in the overall inflammatory responses, such as antigen presentation, leukocyte infiltration, and cytokine production [67,68,69]. In our work, we observed that ocular PM exposure increased the phosphorylation of MAPKs (p38, JNK, and ERK) and subsequently elevated phosphorylation levels of IκBα and NFκB in a PM dose-dependent manner (Figure 2). Our findings clearly demonstrated that ocular PM exposure increased ocular inflammation in ARPE-19 cells by activating MAPK/NFκB signalling and cytokine mRNA expression.

In addition, ocular PM exposure elevated not only inflammatory responses but also ER stress in ARPE-19 cells. Along with its role in protein and lipid synthesis, the ER is also a major quality control center for newly synthesized peptides, as it monitors and modifies folding accordingly or discards misfolded proteins. Moreover, the ER regulates [Ca^2+^]_i_ levels. The ER stress response is triggered by various pathological stimuli such as calcium depletion, hypoxia, oxidative stress, or the production of non-functional proteins, resulting in the accumulation of misfolded or unfolded proteins in the ER lumen [70,71]. Under ER stress conditions, cells activate UPR as a protective signal to maintain homeostasis by three distinct mechanisms: (1) decrease of new protein synthesis by halting protein entry into the ER, (2) increase in protein folding activity, and (3) clearance of misfolded proteins by secretion into the cytosol for ubiquitination and subsequent degradation. However, prolonged ER stress may result in cell death [72,73]. The UPR is initiated with the dissociation of the ER chaperone BiP from three ER membrane sensors (PERK, IRE1α, and ATF6). Activated PERK phosphorylates eIF2α, which halts overall protein translation. Next, phosphorylated eIF2α selectively upregulates ATF4, which translocates to the nucleus and activates proapoptotic CHOP in the nucleus, among other UPR targets. Activated IRE1α splices XBP-1 and controls UPR target genes such as chaperones and ER-associated degradation (ERAD). The IRE1α also binds tumor necrosis factor receptor-associated factor 2 (TRAF2), leading to the recruitment of the IκB kinase (IKK), which phosphorylates IκBa and activates NFκB that subsequently promotes cytokine production [74]. The IRE1α-TRAF2 complex may also recruit apoptosis signal-regulating kinase 1 (ASK1), which upregulates JNK and p38 [75]. Activated ATF6 translocates to the Golgi where it is cleaved and then enters the nucleus to activate UPRs. In the present study, we observed the induction of two PM-inducible UPR pathways: (1) the PERK-eIF2a-ATF4-CHOP axis, and (2) the IRE1α-XBP-1 axis, with concomitant activation of BiP as well (Figure 7A–H). These data are consistent with the upregulation observed in the mRNA expression for *BiP*, *CHOP*, and *XBP-1* (Figure 5). Moreover, ocular PM exposure significantly elevated [Ca^2+^]_i_ levels (Figure 7I), which may trigger hypoxia and angiogenesis. In our experimental setting, the hypoxic markers *ANKRD37* and *VEGFα* were increased significantly following retinal PM exposure (Figure 6B,C).

## 5. Conclusions

In conclusion, our current work demonstrated that ocular PM exposure led to ocular inflammation and ER stress in ARPE-19 cells, which in turn promoted ER stress-related responses, such as UPR. Our findings could provide useful insight into clinical and non-clinical investigations that focus on the mechanisms by which ocular PM exposure may trigger inflammatory responses in the retina, and can lend further support in linking PM with the pathophysiology of the visual system.

Taken together, our findings corroborated our initial hypothesis that ocular PM exposure induces ER stress and subsequent hypoxic adaptation responses, which also include upregulation of UPR-related pathway components.

## Figures and Tables

**Table 1:** Antibodies used for western blot analysis.

Antibody	Full Name	Dilution Factor	Catalog Number
Primary antibodies			
p-p38	phosphorylated p38 mitogen-activated protein kinases	1:1000	CST (#4511)
t-p38	total p38 mitogen-activated protein kinases	1:1000	CST (#8690)
p-JNK	phosphorylated c-Jun N-terminal kinase	1:500	CST (#4668)
t-JNK	total c-Jun N-terminal kinase	1:1000	CST (#9252)
p-ERK	phosphorylated extracellular signal-regulated kinases	1:1000	CST (#4370)
t-ERK	total extracellular signal-regulated kinases	1:1000	CST (#4695)
p-NFκB	phosphorylated nuclear factor kappa B, p65	1:500	CST (#3033)
t-NFκB	total nuclear factor kappa B, p65	1:1000	CST (#8242)
p-IκBα	phosphorylated NFκB inhibitor alpha	1:1000	CST (#2859)
t-IκBα	total NFκB inhibitor alpha	1:1000	CST (#4814)
PERK	protein kinase R-like endoplasmic reticulum kinase	1:200	SCB (#SC-377400)
p-eIF2α	phosphorylated eukaryotic translation initiation factor 2 alpha	1:1000	CST (#9721)
t-eIF2α	total eukaryotic translation initiation factor 2 alpha	1:1000	CST (#5324)
ATF4	activating transcription factor 4	1:1000	CST (#11815)
CHOP	CCAAT/enhancer-binding protein homologous protein	1:1000	CST (#2895)
IRE1α	inositol-requiring enzyme 1 alpha	1:1000	CST (#3294)
XBP-1s	spliced X-box binding protein 1	1:1000	CST (#83418)
BiP	binding of immunoglobulin protein	1:1000	CST (#3183)
GAPDH	glyceraldehyde-3-phosphate dehydrogenase	1:2000	SCB (#SC-47724)
Secondary antibodies			
anti-rabbit-HRP anti-mouse-HRP		1:3000 1:2000	CST (#7044) CST (#7076)

CST; Cell Signaling Technology, Danvers, MA, USA, HRP; horseradish peroxidase, SCB; Santa Cruz Biotechnology, Dallas, TX, USA.

**Table 2:** Primer sequences.

Transcript	Forward Primer (5′ to 3′)	Reverse Primer (5′ to 3′)
*TNFα* *IL-1β* *IL-6* *MCP-1* *BiP* *CHOP* *XBP-1* *ZO-1* *VEGFα* *36B4*	GGC AGT CAG ATC ATC TTC TCG CTC GCC AGT GAA ATG ATG GCT CTT CTC CAC AAG CGC CTT C CCA GAT GCA ATC AAT GCC C CAC AGT GGT GCC TAC CAA G CTC CCA GAG CCC TCA CTC TC TCA CCC CTC CAG AAC ATC TC CGA GTT GCA ATG GTT AAC GGA CTA CCT CCA CCA TGC CAA GT GAA GGC TGT GGT GCT GAT G	GGT TTG CTA CAA CAT GGG CTA GTC GGA GAT TCG TAG CTG GAT CAG GCA ACA CCA GGA GCA TGG TCT TGA AGA TCA CAG CT AGC AGG AAT TCC AGT CAG A TGC TTG AGC CGT TCA TTC TC ACT GGG TCC AAG TTG TCC AG TCA GGA TCA GGA CGA CTT ACT GG GCA GTA GCT GCG CTG ATA GA GTG AGG TCC TCC TTG GTG AA

## Data Availability

The data presented in this study are available from the corresponding authors upon request.
